# Clinical application of single‐molecule optical mapping to a multigeneration FSHD1 pedigree

**DOI:** 10.1002/mgg3.565

**Published:** 2019-01-21

**Authors:** Qian Zhang, Xueqin Xu, Lirong Ding, Huanzheng Li, Chengyang Xu, Yuyan Gong, Ying Liu, Ting Mu, Don Leigh, David S. Cram, Shaohua Tang

**Affiliations:** ^1^ Zhejiang Provincial Key Laboratory of Medical Genetics, School of Laboratory Medicine and Life Sciences Wenzhou Medical University Wenzhou China; ^2^ Wenzhou Key Laboratory of Birth Defects Wenzhou Central Hospital Wenzhou China; ^3^ Berry Genomics Corporation Beijing China; ^4^ The First Hospital of Kunming Kunming China

**Keywords:** 4qA disease alleles, D4Z4 repeats, FSHD1, single‐molecule optical mapping, Southern blot hybridization

## Abstract

**Introduction:**

Facioscapulohumeral muscular dystrophy 1 (FSHD1) is a relatively common autosomal dominant adult muscular dystrophy with variable disease penetrance. The disease is caused by shortening of a D4Z4 repeat array located near the telomere of chromosome 4 at 4q35. This causes activation of a dormant gene *DUX4*, permitting aberrant DUX4 expression which is toxic to muscles. Molecular diagnosis of FSHD1 by Southern blot hybridization or FISH combing is difficult and time consuming, requiring specialist laboratories. As an alternative, we apply a novel approach for the diagnosis of FSHD1 utilizing single‐molecule optical mapping (SMOM).

**Methods:**

Long DNA molecules with BssS1 enzyme marking were subjected to SMOM on the Bionano Genomics platform to determine the number of D4Z4 repeats. Southern blot and molecular combing were used to confirm the FSHD1 haplotypes.

**Results:**

In a study of a five‐generation FSHD1 pedigree, SMOM correctly diagnosed the disease and normal haplotypes, identifying the founder 4qA disease allele as having 4 D4Z4 repeat units. Southern blot and molecular combing analysis confirmed the SMOM results for the 4qA disease and 4qB nondisease alleles.

**Conclusion:**

Based on our findings, we propose that SMOM is a reliable and accurate technique suitable for the molecular diagnosis of FSHD1.

## 1 INTRODUCTION

Facioscapulohumeral muscular dystrophy (FSHD) is an autosomal dominant adult muscular dystrophy, with a population incidence of ~1 in 20,000 (Scionti et al., 2012; Tawil, Maarel, Padberg, & Engelen, 2010). The disease is penetrant by the age of 20 and progressively affects the muscles of the upper body, typically the face, shoulder blades, and upper arms but may also result in deafness, retinal failure, or central nervous system disorders (van der Maarel, Tawil, & Tapscott, 2011; Mul, Boogaard, Maarel, & Engelen, 2016). The subtelomeric chromosomal region 4q35, which is associated with the development of FSHD, consists of multiple copies of a 3.3 kb repeat called D4Z4 (Hewitt et al., 1994). Detailed molecular analyses of this region have identified two allelic variations (termed 4qA and 4qB) of which the reduced size 4qA repeat regions are related to disease pathogenesis (Lemmers et al., 2002, 2003). In unaffected individuals, there are generally upwards of 10 copies of the D4Z4 repeat whereas many affected individuals often have fewer than 10 such repeats (Lemmers et al., 2015; Sacconi, Salviati, & Desnuelle, 2015). In the control population, there are also around 1%–2% of individuals who have 4qA alleles with 8–10 repeats, representing a nonpenetrant polymorphic variant (Scionti et al, 2012).

In the normal state, the D4Z4 repeat region is highly methylated and forms heterochromatin (Gatica & Rosa, 2016; Lemmers et al., 2015). Shortening of the D4Z4 array, however, causes the derepression of flanking genes including *DUX4* located distal of the last D4Z4 repeat (Lemmers et al., 2010; Yao et al., 2014). The ectopic expression of DUX4 protein is toxic in muscle tissues and is now thought to be the main causal factor for FSHD (van der Maarel et al., 2011; Richards, Coppee, Thomas, Belayew, & Upadhyaya, 2012). The 4qA/B haplotype of the last D4Z4 repeat is important for the development of FSHD since the telomeric flanking region of D4Z4 in the 4qA allele contains the 3′ UTR of *DUX4* (also called the pLAM region). It is the presence of this polyadenylation signal that allows stable DUX4 expression and subsequent disease manifestation (Richards et al., 2012). In contrast, individuals with shortened 4qB alleles that lack this polyadenylation signal do not manifest the disease since a nonadenylated, unstable transcript is formed. Over 95% of patients displaying symptoms of FSHD1 have shortened 4qA subtelomeric repeat regions, with disease alleles generally having <10 D4Z4 repeats (Sacconi et al., 2015). A different gene, structural maintenance of chromosomes hinge domain 1 *(SMCHD1*), located at 18p11.32, is associated with the less common FSHD2 (Lemmers et al., 2012) but the clinical phenotype is similar in both types of the disease. In FSHD2, mutations in *SMCHD1* result in a marked hypomethylation of chromosome 4 (and chromosome 10), similarly altering the heterochromatin structure as with D4Z4 repeat contraction and thus allowing chromosome 4 to aberrantly express the *DUX4* transcript and subsequently the toxic protein.

Molecular analysis of FSHD1 using available methods is complicated by the length of the repeat structure (D4Z4), even in reduced array form in affected individuals, as well as the variable size of the subtelomeric region of chromosome 4 in different individuals. In addition, chromosome 10 harbors near identical D4Z4 repeats (Zeng et al., 2014) making both simple PCR approaches and classical Southern hybridization analysis difficult to interpret. More recent approaches have used long range PCR or the more technical pulse field gel electrophoresis (PFGE) followed by Southern hybridization (Wijmenga et al., 1993) to identify array changes. These requirements can be problematic when prenatal diagnosis of the condition is requested. Molecular combing based on FISH of stretched DNA molecules has been developed (Nguyen et al., 2011) but it is yet to achieve any significant clinical application.

A simple uniform method that is easily interpreted and can be adopted by laboratories around the world would be advantageous. In this study, we introduce single‐molecule optical mapping (SMOM) as an alternative approach to diagnose the FSHD repeat structure. Several important problems of existing analysis methods that are resolved include differentiation of 4q35 D4Z4 repeats from the 10q26 array, measurement of actual repeat numbers at 4q35, and differentiation of 4qA from 4qB alleles. SMOM is capable of analyzing DNA fragments from hundreds of kilobases to nearly a megabase in size, and here, we demonstrate successful application of our approach by analyzing clinical DNA samples from a well‐characterized multigeneration FSHD1 pedigree.

## 2 MATERIALS AND METHODS

### 2.1 FSHD1 pedigree

**Figure 1:**
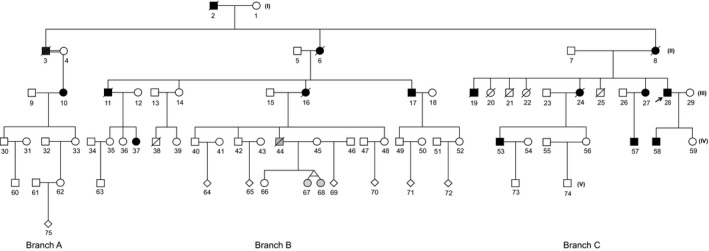
Multigeneration FSHD1 pedigree. The pedigree shows five generations (I, II, III, IV, and V) of three distinct family branches A, B, and C. Black squares and open squares represent affected and nonaffected males, respectively, whereas black circles and open circles represent affected and nonaffected females, respectively. Gray shading for family members 44‐IV, and twin daughters 67‐V and 68‐V indicates a milder FSHD1 phenotype. Diagonal lines through each square or circle signify deceased family members. The black arrow denotes the proband 28

Over the last 10 years, our Prenatal Diagnosis Center in conjunction with hospital physicians and clinical geneticists has been involved in the care of a large FSHD1 pedigree (Figure 1), including the diagnosis, management, and treatment of affected individuals. The research study was approved by the Medical Ethics Committee of Wenzhou Central Hospital (Approval number 2018–02–001), and all selected family members provided written informed consent for further investigation into the genetic basis of their disease. Blood samples were collected in EDTA tubes and the isolated white blood cells used for either extraction of genomic DNA for PFGE/Southern blot and SMOM analyses or, as a source of cells for molecular combing.

### 2.2 Preparation of high molecular weight genomic DNA

For Southern blot, molecular combing, and SMOM analyses, we used an agarose plug extraction method to isolate high molecular weight (MW) DNA as a starting DNA template. High MW DNA was prepared from 2 ml of fresh blood collected in EDTA tubes. DNA isolation was performed following the manufacturer's guidelines (IrysPrep Experienced User Card Human Blood Protocol, Bionano Genomics). In brief, RBC lysis solution (Qiagen) was used to lyse red blood cells and white blood cells (WBCs) were then pelleted by centrifugation. The WBCs were resuspended in cell suspension buffer, embedded into agarose plugs (CHEF Genomic DNA Plug Kit, Bio‐Rad). We used 5–10 × 10^5 ^WBCs per agarose plug. The agarose plugs were washed and then incubated in lysis buffer (Bionano Genomics) plus Puregene Proteinase K (Qiagen) overnight at 50°C. The plugs were then washed with Tris‐EDTA buffer and then either stored at 4°C or proceeded directly to digestion. For digestion, the DNA plug was melted (70°C × 2 min) and then digested at 43°C for 45 min with Agarose (Thermo Fisher Scientific). The recovered DNA was dialyzed for 45 min at room temperature on floating membrane (EMD Millipore, USA) in Tris‐EDTA buffer. The DNA concentration was measured using a Qubit dsDNA BR assay kit (Thermo Fisher Scientific) in a Qubit 3.0 Fluorometer (Thermo Fisher Scientific). Molecular size was determined by pulsed‐field gel electrophoresis. Typically, the size of the high molecular weight DNA ranged from 0.2 to 2 Mb, and >60% of the molecules were over 1 Mb.

### 2.3 Southern blot hybridization

Southern blot hybridization was performed by PFGE of restriction enzyme digested genomic DNA. Fragments were separated by electrophoresis in 0.5× TBE buffer at 6 V/cm for 39 hr. After transfer to a solid phase membrane (GE‐Amersham, HYBOND‐N + membrane), blots were hybridized with biotin‐labeled probes prepared using the North2 SouthTM Biotin Random Prime labeling Kit (Thermo Fischer). Membranes were washed sequentially at RT in 1x SSC prepared in deionized water and then exposed to X‐ray film to identify the target sequences.

For the molecular diagnosis of FSHD1, a series of different restriction enzyme digestions is needed to size the D4Z4 repeat array and discriminate between the 4qA and 4qB alleles (Lemmers et al, 2002). In brief, the first aliquot of genomic DNA was double digested with *Eco*RI and *Hind*III and then blots hybridized with labeled probe p13E‐11, allowing visualization of 4q35 and 10q26 fragments. In parallel, a second aliquot of genomic DNA was triple digested with *Eco*RI, HindIII, and *Bln*I, and blots hybridized with the same‐labeled probe p13E‐11, which only allows visualization of 4q fragments. To identify the 4qA and 4qB alleles, a third and fourth aliquot of genomic DNA was digested with *Hind*III, and blots hybridized with 4qA and 4qB specific probes, respectively.

### 2.4 Molecular combing

Using high MW genomic DNA, DNA fibers were combed and stained with fluorescently labeled probes following the previously published protocols (Nguyen et al., 2017). The fluorescently labeled DNA strands were scanned, and images were analyzed automatically using Combilog® software (Genomic Vision, France).

### 2.5 Single‐molecule optical mapping

High molecular weight DNA was used as the starting input DNA for SMOM analysis. DNA labeling using Nb.BssSI nickase was first performed using the Bionano Prep Labeling NLRS Protocol (Bionano Genomics). Labeled DNA (80 ng) was loaded on a Saphyr chip, and linear molecules were electrophoresed for 24 hr through low‐voltage nanochannels on the Saphyr instrument (Bionano Genomics). During this process, the fluorescently labeled DNA molecules were imaged sequentially across the nanochannels by the Saphyr detectors to produce thousands of high‐resolution images of individual DNA molecules. For data analysis, the Bionano Solve/Access software (Bionano Genomics) was used to align labeled molecules against the labeled reference sequence and to identify signatures of structural variation. First, the raw bins of labeled long DNA molecules were corrected by applying the “autonoise” algorithm to the reference genome to identify noisy regions requiring mapping adjustment. Next, alignment was performed between the rescaled molecules and the reference map using Bionano's custom Refaligner software program. The hg19 reference sequence with a haplotype of 4qB D4Z4(8) was used for discrimination of 4q and 10q molecules (Supporting Information Figure S1). Only the molecules that aligned to the reference chromosome 4q35 region were further collected to generate representative allelic profiles of structural variation to interpret FSHD genotypes. A summary of the SMOM run statistics for the nine selected members of the FSHD1 pedigree that were analyzed in this study is summarized in Supporting Information Table S1.

## 3 RESULTS

### 3.1 Strategy for mapping and differentiation of 4qA and 4qB alleles

The sequence and BssSI labeling pattern of D4Z4 repeat arrays and the upstream 41.6 kb of chromosome 4 and chromosome 10 are almost identical. In order to identify chromosome 4 specific D4Z4 repeat regions, we used the characteristic BssSI label patterns between 69.1 kb and 41.6 kb upstream of the D4Z4 repeat arrays, thus enabling differentiation between chromosome 4 and chromosome 10 (Supporting Information Figure S1). We then filtered the molecules mapped upstream of this threshold (69.1 kb upstream of D4Z4), leaving only the molecules that contain chromosome 4‐specific D4Z4 repeat arrays for analysis.

As there is a BssS1 enzyme site in each of the D4Z4 units, the D4Z4 array would be expected to be labeled every 3.3 kb (the approximate repeat length), thus enabling simple counting of the number of D4Z4 repeats (Figure S1). For the 4qB allele, the terminal D4Z4 repeat contains only the first 570 bp of a complete D4Z4 unit and thus lacks a BssS1 label. In contrast, the terminal D4Z4 repeat of 4qA alleles still contain a BssS1 recognition site, positioned 215 bp distal to the DUX4‐PAS sequence and thus generates a 1.7 kb BssS1 label after the last complete D4Z4 unit. On this basis, the 4qA allele can be readily distinguished from the 4qB allele.

### 3.2 Analysis of a multigeneration FSHD family cohort

Nine surviving members from an extended family cohort of five generations (I‐V) (Figure 1) who had previously undergone detailed clinical examination (Supporting Information Table S2) were selected for SMOM analysis. These included subjects 10‐III (FSHD1), 17‐III (FSHD1), 28‐III (FSHD1), 58‐IV (FSHD1), and 59‐IV (normal) and subject 45‐V (normal) with her twin daughters 67‐V and 68‐V (mild FSHD1) and oldest daughter 66‐V (normal). Among the subjects with confirmed FSHD1, symptom disease severity was associated with progressive age. However, both of the young twin girls 67‐V and 68‐V showed classical signs of early but mild disease onset.

**Figure 2:**
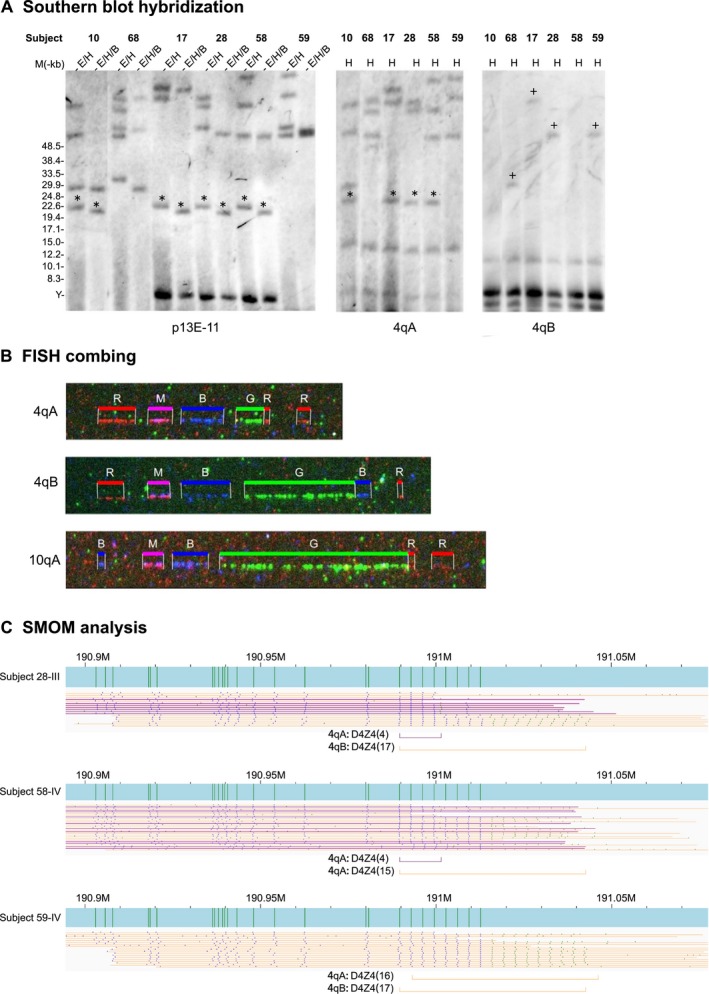
Molecular diagnosis of FSHD1. (a) Southern blot hybridization of selected family members with FSHD1. Membrane‐bound EcoRI/HindIII (E/H), EcoRI/HindIII/BlnI (E/H/B), and HindIII (H) digested genomic DNA from affected and normal family members were hybridized to p13E‐11 (4q35 and 10q26), 4qA, and 4qB labeled probes. 4qA (*) and 4qB (+) fragments. (b) FISH analysis of family member 28‐III (FSHD1). Green signals represent D4Z4 repeat numbers. (c) SMOM analysis of FSHD1 family members. BssSI maps (vertical green bars) of 4qA (purple) and 4qB (orange) alleles for family members 28‐III and 58‐IV (FSHD1) and 59‐IV (normal). The position and number (*n*) of D4Z4 repeats for each allele are indicated by horizontal purple and orange bars

Prior molecular analysis of four surviving subjects with a confirmed diagnosis of FSHD1 was performed by Southern blot hybridization (Figure 2a). All subjects with FSHD1 (10‐III, 17‐III, 28‐III and 58‐IV) had shortened 4qA disease alleles with similar repeat numbers. Molecular combing was also performed on WBCs from subject 28‐III (Figure 2b), showing 5 D4Z4 units for the 4qA allele and 19 D4Z4 units for the 4qB allele, consistent with the molecular diagnosis by Southern blot hybridization. In contrast, twin 68‐V with suspected early FSHD1 disease onset showed a 4qA banding pattern indicative of the longer nondisease‐associated 4qA allele.

**Figure 3:**
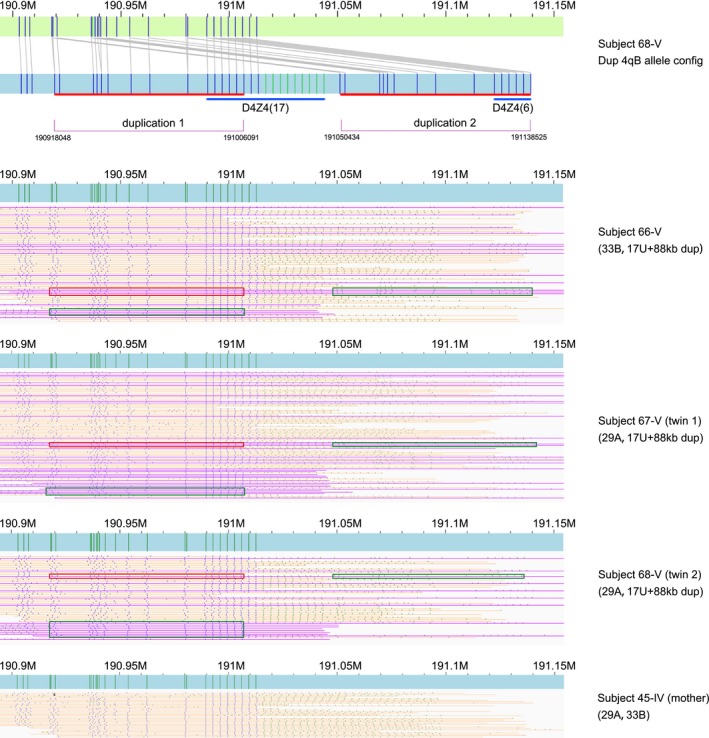
Single‐molecule optical mapping analysis of twins 67‐V and 68‐V with suspected FSHD1. BssSI maps (vertical green bars) of a 4qB allelic variant with a 88 kb duplication (purple) in association with either a normal 4qA or 4qB allele (orange). The duplicated regions are indicated by red and green boxes. The final deduced structure of the allelic variant is represented in the top profile with the duplicated regions indicated by red bars and the altered D4Z4 array repeats by blue bars

SMOM analysis of the nine subjects generated interpretable 4q35 profiles from the aligned reads (Figures 2c and 3) and identified the D4Z4 repeat numbers of both 4qA and 4qB alleles allowing precise determination of the genotypes (Table 1). All subjects with confirmed FSHD1 (10‐III, 17‐III, 28‐III, and 58‐IV) had a copy of a 4qA allele with 4 D4Z4 repeats while subject 10‐III additionally had a 4qA allele with 6 D4Z4 repeats. In contrast, control patient, 59‐IV, had a nondisease 4qA allele of 29 D4Z4 repeats. Based on these genotypes, the 4qA allele with 4 D4Z4 repeats was clearly the founding mutation of the pedigree and likely causal for FSHD1. All SMOM 4qA and 4qB results (Table 1, Figures 2c and 3) were consistent with Southern blot hybridization band patterns (Figure 2a) and FISH combing signals (Figure 2b).

**Table 1:** Molecular diagnosis of FSHD1 by SMOM

Patient ID	Diagnosis	Molecules examined	Complete molecules for diagnosis (number)^a^	Incomplete molecules (number)^b^	Inferred haplotype
10‐III	FSHD1	19	6A(10); 4A(5)	>10U(3); irregular(1)	**4A,** 6A
17‐III(A)	FSHD1	24	34B(9); 4A(6)	4U(2); 1U, 19U, 22U, 26U, 33B, 35U, irregular (each 1)	**4A**, 34B
17‐III(B)		112	4A(37); 34B(30)	31U(5); 30U(4); 27U(3); 20U(3); 12U(5); 6U(7); 4U(2); 29U, 23U, 21U, 19U, 8U, 7U, 5U, 3U(each 1); irregular(8)	**4A**, 34B
45‐IV	Normal	32	33B(13); 29A(6)	29U(2); 24U(2); 20U(1); 18U(2); 11U(1); 8U(2); irregular(3)	33B, 29A
66‐V	Normal	63	17U+88 kb dup(18); 33B(24)	14U(4); 10U(2); 7U(2); 6U(6); 21U, 17U, 11U, 4U, 3U, 1U, irregular(each 1)	33B, 17U+88 kb dup(contains 6B)
67‐V	Features of FSHD1	87	17U+88 kb dup(31); 29A(24)	20U(2); 18U(2); 11U(2); 8U(2); 6U(13); 27U, 24U, 15U, 13U, 7U, 5U, 3U, 2U(each 1); irregular(3)	29A, 17U+88 kb dup(contains 6B)
68‐V(A)	Features of FSHD1	25	17U+ 88 kb dup(9); 29A(7)	17U(2); 1U(2); 17U(2); 6U, 9B, 19U (each 1)	29A, 17U+88 kb dup(contains 6B)
68‐V(B)		47	17U+88 kb dup(16); 29A(6)	17U(7); 8U(2); 6U(7); 2U(2); 15U, 9U, 7U, 4U, 3U (each 1); irregular(2)	29A, 17U+88 kb dup(contains 6B)
28‐III	FSHD1	17	4A(7); 17B(6)	4U(2); 3U(1); irregular(1)	**4A**, 17B
58‐IV	FSHD1	28	4A(11); 15A(5)	1U(2); 16A(2); 16U, 15U, 13U, 12U, 10U, 9U, 4U, irregular (each 1)	**4A, **15A
59‐IV	Normal	19	17B(10); 16A(4)	4U, 5U, 9U (each 1); irregular(2)	16A, 17B

a Molecules nU long enough to span the whole D4Z4 array;

b Molecules nU not long enough to span the whole D4Z4 repeat array, broken downstream of the D4Z4 units. Thus (A) and (B) represent duplicate samples.

Unexpectedly, twins 67‐V and 68‐V with suspected early‐onset FSHD1 did not harbor the founding disease 4qA allele with 4 D4Z4 repeats (Table 1, Figure 3). Instead, SMOM detected a 4qA allele of 29 D4Z4 repeats and a structural variation of the 4qB allele comprising an 88 kb duplicated region whereby the first duplicate contained 17 D4Z4 repeats at the proximal end and the second duplicate contained 6 D4Z4 repeats at the distal end, which we designated 4qB duplication (dup). Analysis of the older sibling 66‐V, who as yet has no signs of disease onset, also revealed the 4qB dup and a standard 4qB allele.

To examine the inheritance and origin of the 4qB dup allelic variant, parental analysis was undertaken using the maternal DNA since the father (44‐IV) was already deceased. By SMOM analysis, 4qB dup was not present in the mother (45‐IV) (Table 1) who had a normal phenotype, indicating that the variant allele was of paternal origin. In this family's medical history, the father was reported to have clinically mild FSHD symptoms that were, however, atypical of the bigger family pedigree. Since he was the source of the 4qB dup allele detected by SMOM, if the clinical descriptions of the twins is correct, it appears that he transmitted a 4qB allele that results in a disease associated with a milder, potentially variable form of FSHD in this family pedigree.

## 4 DISCUSSION

We have demonstrated that Bionano SMOM in the hands of an experienced laboratory is an appropriate and straightforward tool to confirm FSHD1 diagnosis. The judicious use of BssS1 enzyme as the nicking enzyme gave a simplified one‐step approach to distinguish chromosome 4 D4Z4 repeats from those of chromosome 10 and at the same time enable D4Z4 repeat counts and discernment of the 4qA and 4qB alleles from each other. In this study, application of SMOM generated unique, complex maps of the 4q35 region with a significant number of single‐molecule events, allowing enumeration of D4Z4 repeats along with distinction between the 4qA and 4qB alleles. Alignments produced allelic profiles whereby the map signature pattern was virtually identical for each allele. Further, SMOM provided an accurate molecular diagnosis of FSHD, deriving haplotypes consistent with gold standard Southern blotting. Moreover, SMOM results were reproducible, with identical 4q map signatures in duplicate 17‐III and 68‐V DNA samples (Table 1). During the course of our study, two groups have also independently demonstrated the applicability of SMOM on small cohorts of unrelated patients, showing the power of the technology for precisely identifying the disease 4qA and 4qB allelic status (Mitsuhashi et al., 2017; Yi et al., 2018).

Single‐molecule optical mapping analyses of the 5‐generation pedigree identified the founding disease mutation involving a 4qA allele with four D4Z4 repeats. All subjects with this mutation had early‐onset FSHD1 with symptoms progressively worsening with age. Interestingly, in one branch of the pedigree, we revealed a structural variant of the FSHD1 region involving an apparent de novo duplication event. Based on maternal (45‐IV) 4q35 patterns, we tentatively identified the 4qB duplication as belonging to father 44‐IV, who appears to have passed on to both his first daughter and his twin daughters, an allelic variant. So far, only the younger twins show any clinical signs of apparent FSHD1 onset, which appears to be clinically manifesting as a milder form of FSHD1. Therefore, there is a possibility that this structural aberration, in the form of a duplication event, may have generated an unusual disease‐causing 4qB allele. However, a previous molecular study of 4q35 allelic variants associated with FSHD1 only reports the participation of the 4qA allele variants in disease causation (Lemmers et al, 2002). Further clinical studies are ongoing to monitor for any disease onset in daughter (66‐V) as well as any further disease progression in the twin daughters (68‐V and 69‐V), to determine whether this structural variation in the 4qB allele is associated with a mild FSHD1 phenotype.

In summary, the SMOM procedures we developed and validated are relatively simple and achievable by most operational molecular laboratories and so this form of genome mapping broadens the options for FSHD1 disease diagnosis. Also, although not performed in this study, the moderate sample requirements and short time frame of SMOM to analysis compared to Southern hybridization make it a possible option for potential prenatal application, particularly for urgent requests. Further, as demonstrated in this study, SMOM has potential to identify structural variants such as deletions, duplications, or rearrangements of the locus, which may help to better explain the phenotype in some individuals. While it is still early days in the application of this new technology and costs may be currently prohibitive for general routine application, the indications are there that SMOM represents an advance in both FSHD1 diagnosis confirmation and has future practical potential in the modern molecular diagnostic laboratory.

## CONFLICT OF INTEREST

Y.G., Y.L., T.M., D.L., and D.S.C. are employees of Berry Genomics Corporation. None of the authors holds any stocks or bonds in the company. The other authors declare no conflict of interest.
