# Supplementary material for: Clinical application of single‐molecule optical mapping to a multigeneration FSHD1 pedigree
Source: Mol Genet Genomic Med. 2019 Jan 21;7(3):e565. doi: 10.1002/mgg3.565 (PMC6418370; doi:10.1002/mgg3.565)
Supplement: Supplementary file 1 [file MGG3-7-na-s001.pdf]

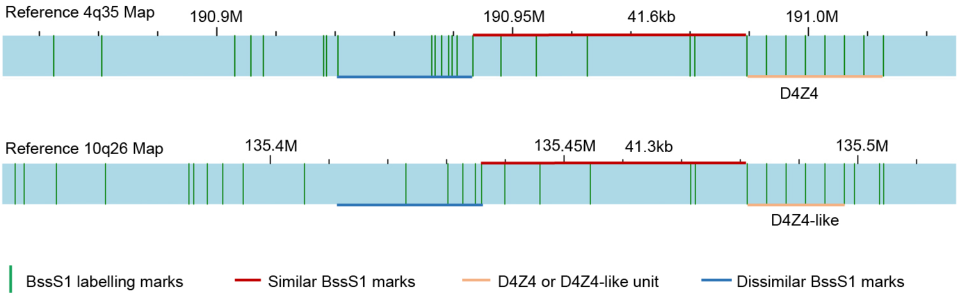

Supplemental figure 1. Differentiation of 4q35 and 10q26 terminal regions by SMOM. BssS1 profiles of the homologous 4q35 and 10q26 telomeric regions. The proximal chromosome segments are unique and distinguish 4q35 from 10q26 whereas the distal region with D4Z4/D4Z4-like repeats are highly similar. The haplotype of the hg19 reference sequence is 4qB D4Z4(8).
