# Supplementary material for: Clinical application of single‐molecule optical mapping to a multigeneration FSHD1 pedigree
Source: Mol Genet Genomic Med. 2019 Jan 21;7(3):e565. doi: 10.1002/mgg3.565 (PMC6418370; doi:10.1002/mgg3.565)
Supplement: Supplementary file 2 [file MGG3-7-na-s002.docx]

**Supplementary Table 1: Clinical features of selected members of FSHD1 pedigree**

| Clinical Parameter/ symptom | Family Branch A | Family Branch B | | | | | | Family Branch C | | |
| --- | --- | --- | --- | --- | --- | --- | --- | --- | --- | --- |
|  | 10-III | 17-III | 44-IV | 45-IV | 66-V | 67-V | 68-V | 28-III | 58-IV | 59-V |
| Age of disease onset | 30 yr | 35 yr | 10 yr | - | - | 14 yr | 14 yr | 15 yr | 15 yr | - |
| Age of clinical examination | 76 yr | 69 yr | Deceased | 42 yr | 20 yr | 15 yr | 15 yr | 50 yr | 26 yr | 14 yr |
| Lower limb weakness | Severe (Dependent  on wheelchair) | Severe (Dependent on [walking](C:/Users/Administrator/AppData/Local/youdao/dict/Application/7.5.2.0/resultui/dict/?keyword=walking)[stick](C:/Users/Administrator/AppData/Local/youdao/dict/Application/7.5.2.0/resultui/dict/?keyword=stick)) | Mild | None | None | None | None | None | None | None |
| Upper limb weakness | Mild | Moderate | None | None | None | None | None | Moderate | Mild | None |
| Shoulder asymmetry, wing-like | Mild | Mild | None | None | None | Mild | Mild | Severe | Moderate | None |
| Muscular atrophy | Moderate | Moderate | Moderate | None | None | None | None | Mild | None | None |
| Myalgia | None | None | None | None | None | None | None | None | None | None |
| Muscle jumping | None | None | None | None | None | None | None | None | None | None |
| Muscle fatigue | Mild | Mild | None | None | None | None | None | Mild | None | None |
| Tendon reflexes | Normal | Normal | Normal | Normal | Normal | Normal | Normal | Normal | Normal | Normal |
| Swallowing disorder | None | None | Mild | None | None | None | None | None | None | None |
| Speech disorder | None | None | None | None | None | None | None | None | None | None |
| Vision, hearing loss | None | None | None | None | None | Mild  (short sight) | Mild  (short sight) | None | None | None |
| Palpitation and shortness of breath | None | None | None | None | None | None | None | Mild | None | None |
| Chest X-ray | ND | ND | ND | ND | ND | ND | ND | Normal | ND | Normal |
| Clinical diagnosis | FSHD1 | FSHD1 | Features of FSHD1 | Normal | Normal | Features of FSHD1 | Features of FSHD1 | FSHD1 | FSHD1 | Normal |
| Molecular diagnosis | FSHD1 | FSHD1 | Unknown | Normal | Normal | Altered 4qB D4Z4  (FSHD1) | Altered 4qB D4Z4  (FSHD1) | FSHD1 | FSHD1 | Normal |
