# Supplementary material for: Clinical application of single‐molecule optical mapping to a multigeneration FSHD1 pedigree
Source: Mol Genet Genomic Med. 2019 Jan 21;7(3):e565. doi: 10.1002/mgg3.565 (PMC6418370; doi:10.1002/mgg3.565)
Supplement: Supplementary file 3 [file MGG3-7-na-s003.docx]

**Supplementary Table 2: Run statistics for SMOM**

| **Sample ID** | **Total DNA**  **>=150kbp**  **Gbp** | **Avg. N50 (>=150kbp)**  **Mbp** | **Avg. N50 (>=20kbp)**  **Mbp** | **Label Density**  **(/100kbp)** | **Map Rate (%)** | **Effective Coverage** | **False Positive (%)** | **False Negative (%)** |
| --- | --- | --- | --- | --- | --- | --- | --- | --- |
| 10-III | 162 | 0.330 | 0.255 | 9.8 | 71.1 | 37.2 X | 9.4 | 9.9 |
| 17-III(A) | 332 | 0.326 | 0.252 | 9.2 | 73.6 | 79.0 X | 6.6 | 13.5 |
| 17-III(B) | 471 | 0.340 | 0.287 | 11.4 | 80.9 | 123.2 X | 9.8 | 9.2 |
| 45-IV | 303 | 0.337 | 0.269 | 11.4 | 85.2 | 83.4 X | 10.3 | 5.5 |
| 66-V | 333 | 0.312 | 0.254 | 10.2 | 80.8 | 86.9 X | 4.9 | 9.1 |
| 67-V | 396 | 0.308 | 0.250 | 11.0 | 78.9 | 100.9 X | 5.1 | 7.9 |
| 68-V(A) | 230 | 0.324 | 0.259 | 9.1 | 73.0 | 54.3 X | 7.8 | 7.0 |
| 68-V(B) | 199 | 0.368 | 0.276 | 11.5 | 76.2 | 48.9 X | 5.2 | 5.2 |
| 28-III | 303 | 0.316 | 0.243 | 9.2 | 82.9 | 81.1 X | 8.1 | 10.0 |
| 58-III | 329 | 0.308 | 0.241 | 10.7 | 85.9 | 91.4 X | 9.0 | 7.9 |
| 59-IV | 296 | 0.333 | 0.272 | 9.9 | 83.2 | 79.6 X | 8.2 | 10.6 |

Avg. N50 (>=150kbp): The average point of half of the mass of the DNA molecule longer than 150K.

Avg. N50 (>=20kbp): The average point of half of the mass of the DNA molecule longer than 20K.

Label Density (/100kbp): The number of labeled sites per 100Kb nucleotides of DNA.

Map Rate (%): Molecules showing high similarity to reference as percentage of 'N Molecules'.

Effective Coverage: Effective coverage depth, based on the Map Rate (%) rather than all the data.

False Positive: The average percentage of molecule labels absent in the reference map.

False Negative: The average percentage of reference labels absent in the aligned molecules.
